# An open-access database of infectious disease transmission trees to explore superspreader epidemiology

**DOI:** 10.1371/journal.pbio.3001685

**Published:** 2022-06-22

**Authors:** Juliana C. Taube, Paige B. Miller, John M. Drake

**Affiliations:** 1 Department of Mathematics, Bowdoin College, Brunswick, Maine, United States of America; 2 Odum School of Ecology, University of Georgia, Athens, Georgia, United States of America; 3 Center for the Ecology of Infectious Diseases, University of Georgia, Athens, Georgia, United States of America; Imperial College London, UNITED KINGDOM

## Abstract

Historically, emerging and reemerging infectious diseases have caused large, deadly, and expensive multinational outbreaks. Often outbreak investigations aim to identify who infected whom by reconstructing the outbreak transmission tree, which visualizes transmission between individuals as a network with nodes representing individuals and branches representing transmission from person to person. We compiled a database, called OutbreakTrees, of 382 published, standardized transmission trees consisting of 16 directly transmitted diseases ranging in size from 2 to 286 cases. For each tree and disease, we calculated several key statistics, such as tree size, average number of secondary infections, the dispersion parameter, and the proportion of cases considered superspreaders, and examined how these statistics varied over the course of each outbreak and under different assumptions about the completeness of outbreak investigations. We demonstrated the potential utility of the database through 2 short analyses addressing questions about superspreader epidemiology for a variety of diseases, including Coronavirus Disease 2019 (COVID-19). First, we found that our transmission trees were consistent with theory predicting that intermediate dispersion parameters give rise to the highest proportion of cases causing superspreading events. Additionally, we investigated patterns in how superspreaders are infected. Across trees with more than 1 superspreader, we found preliminary support for the theory that superspreaders generate other superspreaders. In sum, our findings put the role of superspreading in COVID-19 transmission in perspective with that of other diseases and suggest an approach to further research regarding the generation of superspreaders. These data have been made openly available to encourage reuse and further scientific inquiry.

## Abstract 2

This study compiles and standardizes reported infectious disease transmission trees to analyze trends in superspreader frequency and generation; these data support theories that intermediate dispersion parameters give rise to the highest proportion of cases causing superspreading events, and that superspreaders generate other superspreaders.

## Introduction

In the past 20 years, emerging and reemerging infectious diseases have caused large, deadly, and expensive multinational outbreaks of SARS-CoV (Severe Acute Respiratory Syndrome (SARS)), Zika, Ebola, measles, and SARS-CoV-2 (Coronavirus Disease 2019 (COVID-19)). During outbreaks, public health officials conduct routine investigations to identify who infected whom and reconstruct the transmission tree. Transmission trees visualize transmission between cases as directed networks with nodes representing individuals and edges representing transmission from person to person. Transmission trees are typically reassembled by case-finding, contact-tracing, and detailed epidemiological interviews, followed sometimes by genome sequencing and/or probabilistic reconstruction, where the probability that one case infected another is calculated for each pair of cases [1,2]. These investigations are costly but valuable because transmission trees are information rich, including details about the settings of transmission and variation in number of secondary infections.

When published, transmission trees are shown and described in a variety of formats that makes them difficult to compare across outbreaks, let alone pathogens. Some are presented graphically using a number of different symbols and colors, or are buried in the text, making connections hard to piece together. The primary goal of this project was to create a standardized database of transmission trees that is easily accessible and analyzable. We hope that the OutbreakTrees database allows scientists and public health officials to take further advantage of outbreak investigations and their findings.

One phenomenon that is apparent in transmission trees is superspreading, which is important to the propagation patterns of several infectious diseases [3]. Lloyd-Smith and colleagues [3] quantitatively defined superspreaders as cases that cause more secondary infections than the 99th percentile of a Poisson(*R*_0_) distribution, where *R*_0_ is the basic reproductive number, or average number of secondary infections per case. Lloyd-Smith and colleagues [3] also conceptualized the offspring distribution (i.e., the number of infections caused by each infected individual) as a negative binomial distribution with dispersion parameter *k* and mean *R*. Large values of *k* denote little variation in number of secondary infections caused by each case, while small values of *k* (*k*<1) correspond to high heterogeneity in the offspring distribution. It was hypothesized that intermediate dispersion parameters between 0.1 and 1, depending on *R*, would give rise to the highest proportion of cases causing superspreading events [3].

Lloyd-Smith and colleagues’ theory on superspreading assumes stability of *R* and *k* over the course of an outbreak. In reality, most outbreaks are subject to control measures. These control measures, as well as changes in behavior, can reduce disease transmission and disperse the offspring distribution, thus leading to shifts in *R* and *k* from their pre-control values, as explored by [3]. Given information on the timing of control measures, parameter values can be compared before and after controls were imposed. In the absence of this information, we propose that a comparison of parameter values in the first versus second half of a transmission tree indicates the effect of control measures and behavior changes on a given transmission tree.

While previous work has characterized the biological and social factors that give rise to superspreading events [4], how superspreaders are generated (i.e., who spreads to superspreaders) is poorly understood. In 2020, Beldomenico [5] suggested that the generation of superspreaders may be linked to biological patterns in initial viral dosage: If individuals with unusually high viral shedding cause those they infect to also have high viral shedding, then cases infected by superspreaders may be disproportionately likely to be superspreaders themselves. Another possibility is that superspreaders may be more likely to engage in riskier behavior (such as attending large gatherings or not taking precautionary measures) making them more likely to infect others with similar behavior. This behavioral heterogeneity may be a larger contributor to superspreader generation than biological heterogeneity [6]. We investigate this issue using transmission tree data, hypothesizing that superspreaders will be more likely to be infected by other superspreaders than non-superspreading cases.

## Methods

### Data

Transmission trees were collected by searching Google Scholar, Scopus, PubMed, and Google Images for published literature containing graphs of transmission trees or written accounts of transmission events. We used the following terms to find papers containing transmission tree information: “transmission AND (tree OR network OR chain) AND (outbreak OR disease),” “outbreak investigation,” “contact tracing,” “case report,” and “transmission tree outbreak reconstruction.” We also used the bibliographies of other papers (e.g., [3]) to find more references. With the emergence of COVID-19, we expanded our search for transmission trees to include news articles and preprints (e.g., medRxiv.org). For COVID-19, many of the trees were identified with an online database [7]. If trees could not be collected from a public source or if trees did not identify single infectors for each infectee, we contacted the authors of identified documents for further clarification or additional information. We also compiled readily available node attributes reported in the tree source. Attributes available for each tree varied but included age, sex, context of transmission, date of symptom onset, occupation, quarantine status, survival status, location, hospital, ward of hospital or care facility, symptomatic status, duration of exposure to infected individual, whether the edge was probabilistically reconstructed, relationship between individuals, serial interval, immunization status, source of edge (if tree was constructed from 2 sources), and strain or genomic sequence. Articles in languages other than English were translated using Google Translate software.

**Fig 1:**
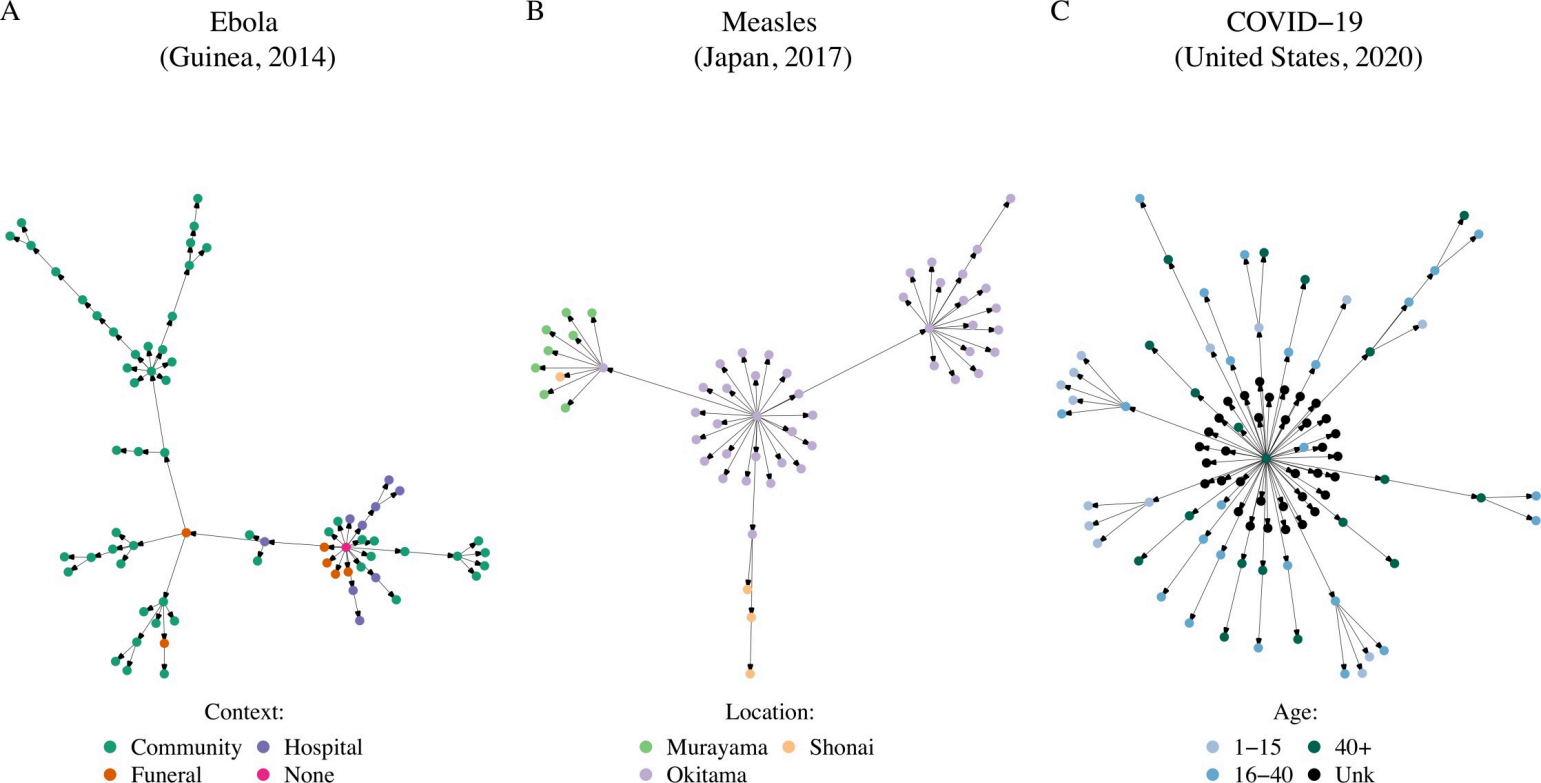
We compiled infectious disease transmission trees from the literature along with reported attribute information. Shown here are example trees in the database. (A) Ebola spread in different contexts [8]. (B) Measles spread in different locations [9]. (C) COVID-19 spread among age classes [10]. Primary sources for transmission trees are available in OutbreakTrees and listed in the Supporting information. OutbreakTrees may be accessed online at http://outbreaktrees.ecology.uga.edu. COVID-19, Coronavirus Disease 2019.

Examples of trees contained in our published database OutbreakTrees are shown in Fig 1.

### Inclusion criteria

For consistency, we required that trees meet the following criteria for inclusion in the database:

- Trees must have contained 2 or more individuals; case studies of isolated infected individuals were excluded.
- Trees must represent outbreaks of directly transmitted infectious diseases in humans; trees describing sexually transmitted, foodborne, vector-borne, or waterborne diseases, as well as diseases in nonhumans (e.g., outbreaks among farm animals [11,12]) were excluded.
- Trees were constructed through epidemiological or probabilistic methods; trees constructed using only genomic or phylogenetic methods were excluded.
- Trees had to report a single infector per infectee (i.e., trees that had multiple possible infectors for any case were excluded). However, if tree topology was unaffected by randomly assigning ambiguous infectors, we included the tree and omitted specific attribute data for the assigned infector.
- Trees were presented as completed investigations in the publication; we excluded trees presented as under ongoing investigation at the time of reporting.

### Data entry

Trees were manually encoded as data.tree [13] objects using relevant information from each source and converted to igraph [14] objects for manipulation and accession. Any assumptions made in entering the tree are listed with the tree in the database (e.g., if an infector is assumed due to nodes obscuring branches or a case of an ambiguous infector assignment). All scripts to compile trees and analyze data are available at http://github.com/DrakeLab/taube-transmission-trees, and tree sources are listed in S1 File. The database is available online at http://outbreaktrees.ecology.uga.edu.

### Data analysis

We demonstrated how OutbreakTrees can be used to address questions about the time dependence of epidemiological parameters and the role of superspreading in infectious disease transmission through 3 different analyses using trees with 20 or more cases and 2 or more generations of spread. We calculated key statistics under 2 contrasting assumptions about outbreak investigation completeness, explained in the Sensitivity analyses section below.

#### Parameter time dependence

Shifts in human behavior or disease control efforts can cause changes in key epidemiological parameters as outbreaks progress [3]. While information on intervention timing was not readily available, we explored how *R*, *k*, and the proportion of cases causing superspreading events varied over time by comparing these values in the first versus second halves of each tree. Excluding the last generation of the tree (composed solely of terminal nodes), we divided each tree into first and second halves by generation. Middle generation nodes were randomly assigned to either the first or second half of the tree. We repeated this process 10 times to account for random variation in the assignment of middle generation nodes and took the mean parameter values over the 10 repetitions. Differences were tested for significance using the Wilcoxon rank test. If population control efforts or human behavior changed transmission dynamics partway through the tree, we expected to see decreases in *R*, *k*, and the proportion of cases causing superspreading events between the first and second halves of a tree [3].

#### Superspreading events across diseases

To evaluate how common superspreading is among different diseases, we focused on 2 tree statistics: (1) the proportion of cases causing superspreading events and (2) the dispersion parameter, *k*. The proportion of cases causing superspreading events was calculated by dividing the number of superspreaders in a tree by the total number of nodes in the tree, where the number of superspreaders was estimated using the Lloyd-Smith and colleagues [3] definition. The dispersion parameter was calculated using maximum likelihood estimation with the fitdistr function from the mass package in R [15] assuming secondary infections followed a negative binomial distribution. Small dispersion parameters indicate more heterogeneous offspring distributions with fewer individuals accounting for the majority of transmission compared with large dispersion parameters. We performed sensitivity analyses for cutoffs of trees with 10 and 30 or more cases.

#### Generation of superspreaders

Next, we investigated patterns in the individuals who infected superspreaders. We calculated the ratio of observed to expected superspreader-superspreader dyads. Superspreader-superspreader dyads occur when 1 superspreader infects another. To determine the expected number of dyads per tree, we calculated the probability that a given edge connects 2 superspreaders. Denoting the number of superspreaders by *s*, number of terminal nodes (nodes that do not cause onward transmission) by *t*, and tree size by *S*, the probability that a node at 1 end of the edge is a superspreader is $\frac{s}{S-t}$, or *p*_1_. Conditional on this first node being a superspreader, the probability that the node on the other end of the edge is a superspreader is $\frac{s-1}{S-t-1}$, or *p*_2_. Then, the joint probability of an edge with superspreaders at either end (a dyad) is *p*_1_·*p*_2_. Given that there are *S-t-1* edges leading to nonterminal nodes in a tree, the expected number of dyads is $(S-t-1)\cdot p_{1}\cdot p_{2}=\frac{\left(S-t-1)s(s-1\right)}{\left(S-t)(S-t-1\right)}$ which simplifies to $\frac{s(s-1)}{S-t}$. Thus, we expect to see $\frac{s(s-1)}{S-t}$ superspreader-superspreader dyads per tree. If generation of superspreaders is not random but tied to characteristics of the infector, we would expect to see large ratios of observed to expected superspreader-superspreader dyads.

#### Sensitivity analyses for tree completeness

We made the assumption that trees in the database depicted complete epidemics, e.g., that all transmission events were documented and that terminal nodes did not transmit disease, yet we know that not all trees in the database are complete (see Limitations section). Recognizing that this is an extreme assumption, we conducted sensitivity analyses of the opposite extreme: Assuming all trees were incomplete, i.e., terminal nodes did transmit disease but these transmission events went unreported. In reality, the database is composed of both types of trees, complete and incomplete, as well as trees somewhere in between (e.g., last generation terminal nodes are not reliable but terminal nodes in earlier generations may be reliable), though we cannot identify which trees fall into which categories. Assuming that trees were complete, we calculated *R*, *k*, and the superspreading cutoff over all nodes in the tree, whereas under the assumption of incompleteness, we calculated *R*, *k*, and the superspreading cutoff by excluding the out-degree (zero) of all terminal nodes in any generation from the offspring distribution. We expect that *R* and *k* estimates will be higher and proportion of cases causing superspreading events estimates lower when we calculate these parameters over only nonterminal nodes than when calculated over all nodes in a tree. Results from our repeated analyses under this alternative set of assumptions can be found in the Supporting information (S1–S3, S6 and S7 Figs).

## Results and discussion

### Database summary statistics

**Fig 2:**
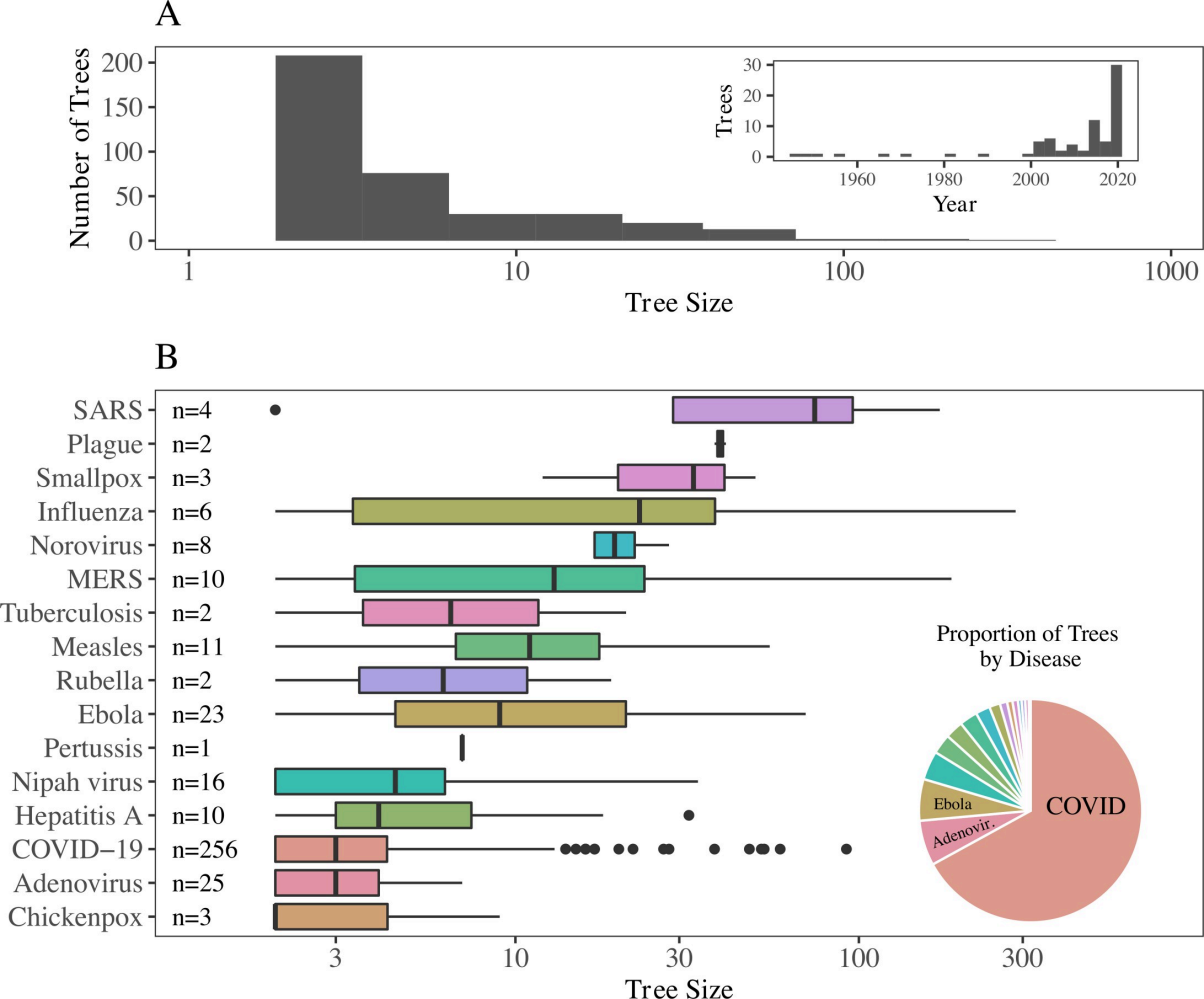
Characteristics of transmission trees in OutbreakTrees. (A) Tree size varies from 2 to 286 with a median of 3 and most trees represent outbreaks taking place in the past 20 years (only trees with 10 or more cases shown in date plot due to large number of small COVID-19 trees from 2020). (B) The largest trees are from H1N1 and SARS outbreaks, while the highest proportion of trees in the database are from outbreaks of COVID-19, followed by adenovirus and Ebola. Tree size axes in both plots are shown on a *log*_10_ scale to better illustrate variation in medium-sized trees. All trees are used in this analysis. The data to reproduce this figure can be found at https://doi.org/10.5061/dryad.nk98sf7w7. COVID-19, Coronavirus Disease 2019; MERS, Middle East Respiratory Syndrome; SARS, Severe Acute Respiratory Syndrome.

Currently, OutbreakTrees includes 382 trees describing 16 directly transmitted infectious diseases (see Fig 1 for examples), most of which are caused by viruses (Fig 2). COVID-19 trees comprise 256, or approximately 67%, of the trees in the database. Trees range in size from 2 to 286 individuals; half are composed of 3 cases or fewer. This database contains data for outbreaks that took place in the years 1946 through 2020. The most common node attributes for trees include context of transmission (work, school, family, etc.), date of symptom onset, sex, and age (Table 1). Due to imperfect investigation or recall, specific attributes are not available for every node in every individual tree (S1 Table).

**Table 1:** List of most common attributes for individuals in trees.

Attribute	Database code	Number of trees
Transmission context	cont	301
Symptom onset	onset	137
Sex	sex	86
Age	age	69
Location	loc	56
Quarantine status	quar	36
Occupation	occp	34
Survival	surv	20

### Analyses

For the following analyses, we use a subset of trees in the database to ensure sufficient sample size for statistical analysis [16]. Specifically, estimates of *R*, the dispersion parameter *k*, the threshold number of secondary infections to be considered a superspreader, and the proportion of cases causing superspreading events for each tree are limited to trees with 20 or more cases and at least 2 generations of spread. There were 39 trees in our database that fit these criteria. The differences in *R* and *k* values depending on our assumptions of tree completeness are shown in S1 and S2 Figs. Note that when we calculate *R* assuming all cases are reported and the infection has died out, then *R* is necessarily <1 (S1 Fig). Applying the Lloyd-Smith and colleagues [3] definition of superspreading with *R*≈1, the superspreading threshold is always more than 4 secondary infections. When we instead assume that a transmission tree is incomplete (i.e., not all cases are reported) and exclude terminal nontransmitting nodes from our calculation of *R*, we observe higher *R* values, and consequently higher superspreading cutoffs that show greater variation across diseases (S1 Fig).

#### Parameter time dependence

**Fig 3:**
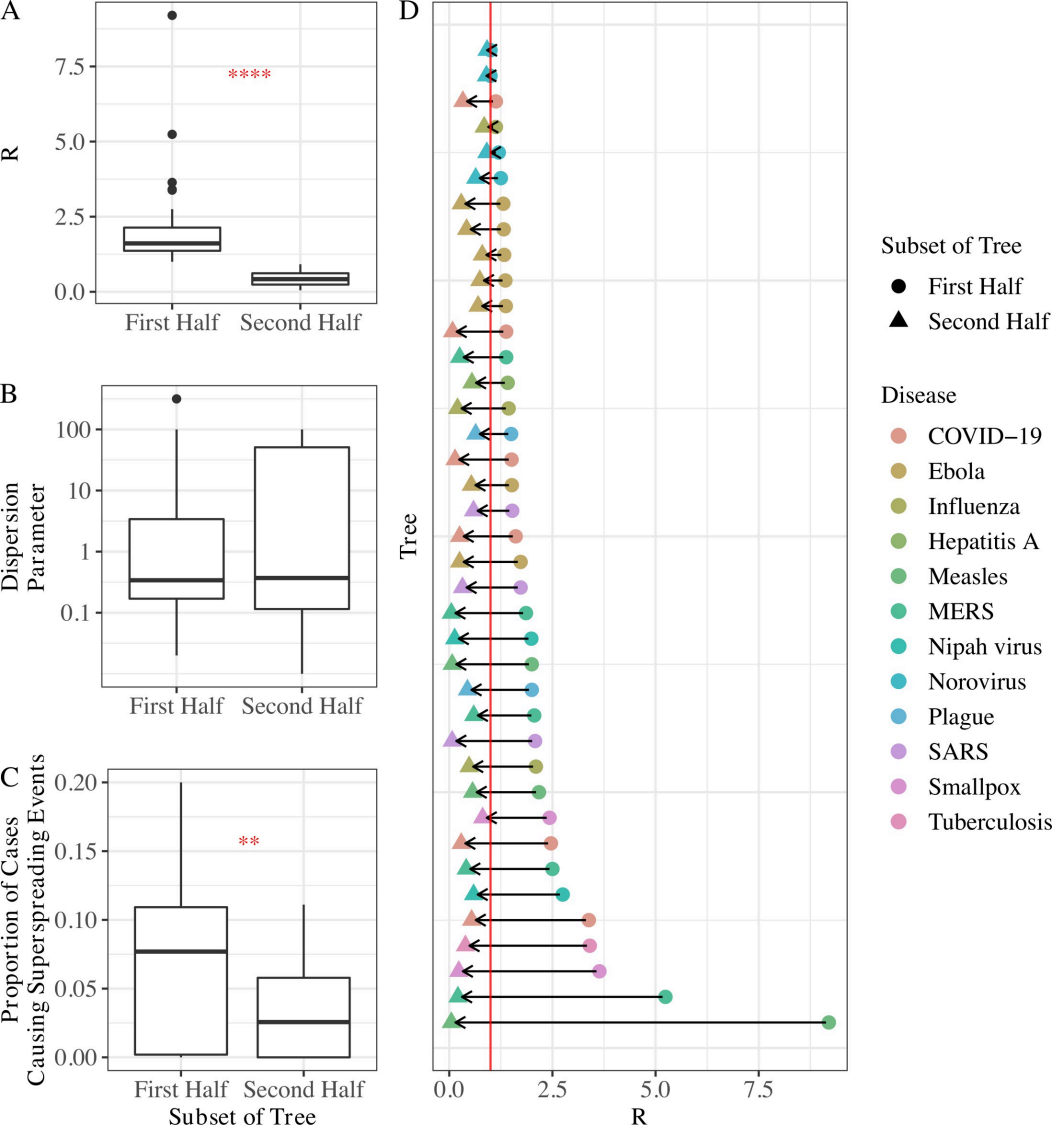
The time dependence of *R*, *k*, and the proportion of cases causing superspreading events. (A) *R* decreased significantly between the first and second halves of transmission trees. (B) *k* did not differ significantly between the first and second halves of transmission trees. Y-axis is on a *log*_10_ scale for visual aid. (C) The proportion of cases causing superspreading events decreased significantly between the first and second halves of transmission trees. (D) Decrease in *R* shown for each tree by disease. *R* was below 1 in the second half of all trees; red line denotes *R* = 1. The Wilcoxon rank test was used for all significance tests (*: *p*≤0.05, **: *p*≤0.01, ***: *p*≤0.001, ****: *p*≤0.0001), and results are shown in red stars. Trees were assumed to be complete and only trees with 20 or more cases and at least 2 generations of spread were used in these analyses. Results assuming tree incompleteness are shown in S3 Fig. The data to reproduce this figure can be found at https://doi.org/10.5061/dryad.nk98sf7w7. COVID-19, Coronavirus Disease 2019; MERS, Middle East Respiratory Syndrome; SARS, Severe Acute Respiratory Syndrome.

We found a significant decrease in *R* (*p*≤0.0001, Wilcoxon rank test) and the proportion of cases causing superspreading events (*p*≤0.01, Wilcoxon rank test) between the first and second halves of transmission trees with 20 or more nodes and 2 or more generations of spread assuming tree completeness (Fig 3A and 3C). The dispersion parameter did not change significantly between the first and second halves of these transmission trees (Fig 3B, Wilcoxon rank test). While all but 3 trees had *R*>1 in the first half of the tree, all trees had *R*<1 in the second half of the tree (Fig 3D). Under the assumption of incomplete trees, all 3 parameters changed significantly between the first and second halves of the trees (S3 Fig); *R* decreased (*p*≤0.0001, Wilcoxon rank test), *k* increased (*p*≤0.01, Wilcoxon rank test), and the proportion of cases causing superspreading events decreased (*p*≤0.001, Wilcoxon rank test). The observed decreases in *R* may be the result of control measures or behavior changes in the affected populations, or could be caused by reporting biases where case follow-up is more robust in earlier generations. Similarly, the decreases in proportion of cases causing superspreading events could be due to control measures, but also superspreaders may be more likely to be identified in earlier generations if superspreading events spur outbreak investigations which may only trace transmission so far back in time. The increase in *k* under an assumption of tree incompleteness contradicts our expectation but may be due to the truncation of the offspring distribution to a minimum of 1 secondary infection when terminal nodes are dropped from our calculations. This truncation may disproportionately affect the second half of a tree with many terminal nodes, decreasing the heterogeneity in the number of secondary infections, and increasing *k*. This analysis informs the following 2 analyses by indicating how frequently our trees may be capturing disease spread after interventions are imposed or behavior changes take place.

#### Superspreading characteristics across diseases

**Fig 4:**
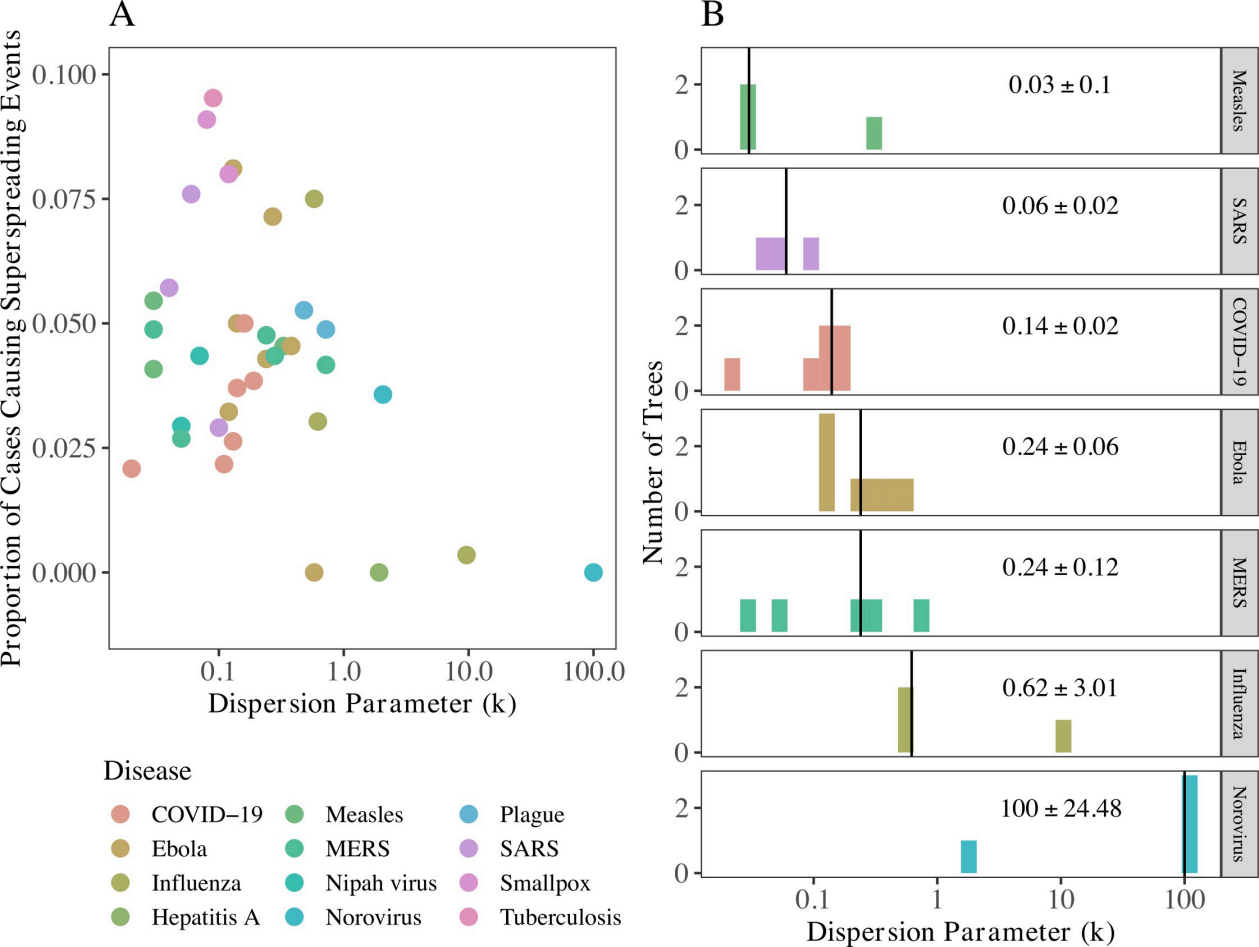
The importance and expected frequency of superspreading across diseases. (A) The highest proportion of cases causing superspreading events is observed at intermediate dispersion parameters, as predicted by theory [3]. (B) Dispersion parameter (*k*) of a negative binomial distribution fit to the offspring distribution of trees by disease (for diseases with at least 3 trees). Lower dispersion parameters are indicative of greater variation in number of secondary infections. Vertical line and value printed in each facet shows the median *k* and standard error for each disease. X-axes are on a *log*_10_ scale in both plots for visual aid. Trees were assumed to be complete and only trees with 20 or more cases and at least 2 generations of spread were used in these analyses. Other size cutoffs are shown in S4 and S5 Figs and results assuming tree incompleteness are shown in S6 Fig. The data to reproduce this figure can be found at https://doi.org/10.5061/dryad.nk98sf7w7. COVID-19, Coronavirus Disease 2019; MERS, Middle East Respiratory Syndrome; SARS, Severe Acute Respiratory Syndrome.

Consistent with theory proposed by [3], intermediate dispersion parameters gave rise to the highest proportion of cases causing superspreading events (Fig 4A). COVID-19 trees had a median dispersion parameter (*k* = 0.14) (Fig 4B) between that of SARS (0.06) and Middle East Respiratory Syndrome (MERS) (0.24). Six diseases had overdispersed offspring distributions (median *k*<1): measles, SARS, COVID-19, Ebola, MERS, and influenza. Norovirus was the only disease with median *k*>1. Dispersion parameter estimates calculated over all nodes tend to be lower than (or at the lower end) of values/ranges in the literature, while estimates calculated excluding all terminal nodes (shown in S6 Fig) tend to be higher than (or at the higher end) of values/ranges in the literature [3,17–31]. Given that our assumptions about tree completeness lie at opposite extremes, we expect the true outbreak dispersion parameters to fall between these extremes, which aligns well with the literature. The most notable exceptions are influenza, which is not typically associated with superspreading (though our median dispersion parameter estimate was less than 1), and norovirus, for which we could not find a previously published dispersion estimate. As observed with some of the large standard errors of *k*, and covered extensively in [16], these estimates are imprecise, especially when based on smaller trees. However, we observe little change in median dispersion parameter estimates or the relationship between dispersion parameter and proportion of cases causing superspreading events when we restrict the analysis to trees with at least 2 generations of spread and 10 or more cases (S4 Fig) or 30 or more cases (S5 Fig). Lack of follow-up in outbreak investigations may result in underreporting of onward transmission, affecting tree offspring distributions, and consequently, estimates of *k*.

#### Generation of superspreaders

**Fig 5:**
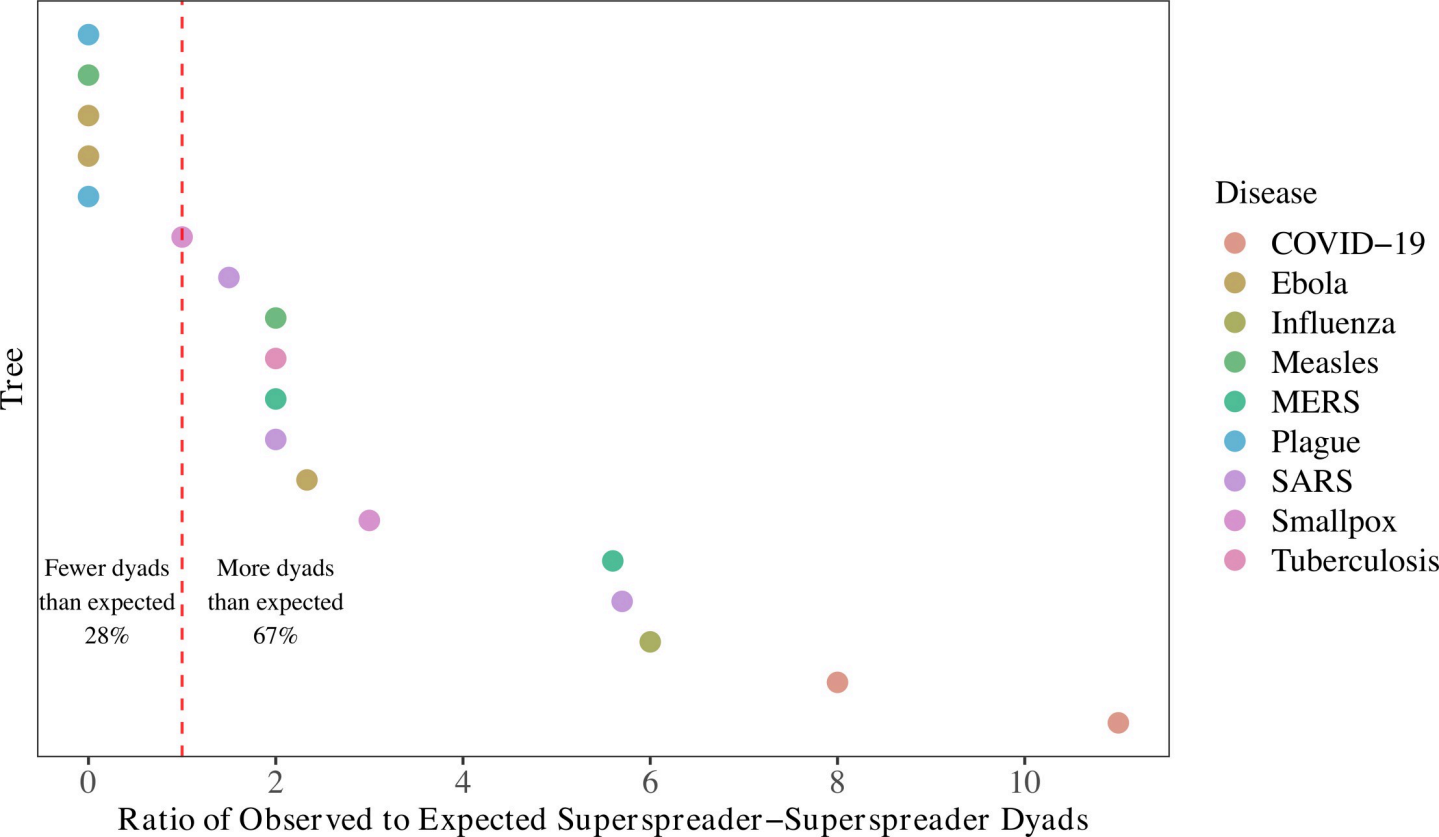
In two-thirds of transmission trees, superspreaders infect superspreaders more often than would be expected by chance. The expected number of superspreader-superspreader dyads was calculated by $\frac{s(s-1)}{S-t}$ for each tree, where *s* is the number of superspreaders in the tree, *t* is the number of terminal nodes (nodes that do not cause onward transmission), and *S* is tree size. Ratios larger than 1 indicate more superspreader-superspreader dyads were observed than would be expected by chance. This analysis was limited to trees with more than 1 superspreader, 20 or more cases, and 2 or more generations of spread. We assumed tree completeness here, but results assuming incompleteness are shown in S7 Fig. The data to reproduce this figure can be found at https://doi.org/10.5061/dryad.nk98sf7w7. COVID-19, Coronavirus Disease 2019; MERS, Middle East Respiratory Syndrome; SARS, Severe Acute Respiratory Syndrome.

The ratio of observed to expected superspreader-superspreader dyads, calculated by enumerating superspreader-superspreader pairs divided by all possible nonterminal infector–infectee pairs, was greater than 1 for 12 of 18 trees, indicating that superspreaders infected other superspreaders more than would be expected by chance in two-thirds of eligible trees (Fig 5). Notably, both COVID-19 trees under consideration had large ratios of observed to expected superspreader-superspreader dyads. (Recall that we expect $\frac{s(s-1)}{S-t}$ dyads in a tree of size *S* with *s* superspreaders and *t* terminal nodes.) Despite most trees in our sample being small—29 of 39 trees have less than 50 cases—our observation of a large number of dyads suggests that this transmission pattern must be common. If we instead assume tree incompleteness, only 4 trees have enough superspreaders to compare ratios of observed to expected dyads (S7 Fig). Though additional information regarding the contexts in which superspreaders are infected would be required to understand these patterns, these results suggest some nonrandomness in generation of superspreaders providing preliminary support for our hypothesis that superspreaders infect other superspreaders.

### Limitations of OutbreakTrees

While OutbreakTrees has allowed us to investigate questions about the nature of superspreading, the database has several limitations. First, trees in the database do not constitute a random nor necessarily representative sample of directly transmitted infectious disease outbreaks. For example, we omitted nearly 100 reported transmission events and trees due to lack of single infector identification, which limits the generalizability of our findings. Furthermore, as shown by Lloyd-Smith and colleagues [3], diseases with larger variation in offspring distributions have a greater chance of extinction. Early superspreading events may prevent extinction by increasing the size from which the outbreak grows and making infection propagation more likely [32]. The probability of detecting an outbreak may also be higher if there is a superspreading event because public health officials are more likely to investigate a cluster than an isolated case. Thus, the trees represented in our database are prone to both selection bias, in which outbreaks are noticed, and publication bias, in which outbreaks are published in an accessible format.

Second, although trees are meant to be complete representations of clusters (see Inclusion criteria), they are typically a subset from a larger chain of transmission events. For example, Ebola was likely only introduced once in the 2014 outbreak in West Africa, yet we have several separate trees because the transmission events could not all be connected. Moreover, outbreak investigations may miss cases, sometimes in random or consistent ways. For example, secondary cases with ambiguous infectors may be more readily attributed to superspreaders than their actual infectors, making it look like superspreaders accounted for more cases than they actually did. Or, as an outbreak continues, later cases may not be investigated in the same depth as earlier generations, underrepresenting the number of secondary infections produced by cases in later generations.

Third, control measures or behavior changes can alter parameters of disease spread in the middle of an outbreak. Due to limited available data, we have not included the timing of these events in the database, but they have the potential to affect every outbreak. For example, interventions may reduce the number and disperse the distribution of secondary infections caused by each individual. The scope of the database also does not include details about how each tree was constructed for publication. Reconstruction methods may be biased in different ways; methods focused on symptomatic cases may miss asymptomatic cases and transmission events. We were mindful of these biases and sought to examine how several key parameters change over the generations in our trees. These limitations should be kept in mind by others using the database for different purposes.

### Usage notes

We have constructed the database so that other research groups may take advantage of this new resource, but we acknowledge that care and understanding of the limitations are required for responsible analyses. Thus, we provide these recommendations for future users to encourage appropriate use and generalizable conclusions. We opted to include small trees in the database for the sake of completeness and to allow for the possibility of minor outbreak analysis in the future (e.g., [33]), but suggest that these smaller trees be excluded if users are seeking to calculate epidemiological quantities (as we did with a size cutoff of 20 individuals in our analyses). We also urge caution in viewing trees as absolute or complete. Several trees in the database are the result of probabilistic reconstruction, and so may represent only one possible way in which transmission may have occurred. Lack of ongoing transmission at the terminal nodes of a tree may be real but also could be due to lack of follow-up or investigation. While conclusions drawn from the database may be biased, they are no more biased than the original inferences drawn from the individual trees which compose the database. With these suggestions in mind, we hope that OutbreakTrees can be used to properly address new questions in the future.

## Conclusions

In summary, we developed OutbreakTrees, an open-access database of infectious disease transmission trees, for research and public health officials. We illustrated how this database can be used to explore questions surrounding superspreader epidemiology, and we calculated a few important parameters for COVID-19 and examined their time dependence. In particular, we estimated the dispersion parameter from transmission trees and the value for COVID-19 was in between that of SARS and MERS. Additionally, our analysis provided tentative support for the theory that superspreaders generate other superspreaders. The development and release of OutbreakTrees highlights the benefits of data sharing and offers a new resource for epidemiologic research.

## Supporting information

S1 File Sources for transmission trees in OutbreakTrees which were used for this analysis. (PDF)

S1 Fig *R* values for each disease varied depending on calculation method. *R* values tended to be highest when calculated over nonterminal nodes and lowest when calculated over all nodes, with estimates based on early generation nodes (root and first generation nodes) falling somewhere in between. Nonterminal node estimates tended to be at the high end of literature values and early generation estimates at the low end, with estimates calculated over all nodes typically far below literature values [20,29,34–44], except for MERS and SARS which had low literature *R* estimates [3,21,30,45]. Analysis was limited to trees with 20 or more cases and at least 2 generations of spread and diseases with at least 3 trees that meet these criteria. The data to reproduce this figure can be found at https://doi.org/10.5061/dryad.nk98sf7w7. COVID-19, Coronavirus Disease 2019; MERS, Middle East Respiratory Syndrome; SARS, Severe Acute Respiratory Syndrome.
(PDF)

S2 Fig Dispersion parameters were consistently higher when calculated over only nonterminal nodes versus all nodes in a tree. Dispersion parameter calculated over all nodes is on x-axis on *log*10 scale; dispersion parameter calculated over all nonterminal nodes is on y-axis on *log*10 scale. Dashed red line is y = x. Analysis was limited to trees with 20 or more cases and at least 2 generations of spread. The data to reproduce this figure can be found at https://doi.org/10.5061/dryad.nk98sf7w7. COVID-19, Coronavirus Disease 2019; MERS, Middle East Respiratory Syndrome; SARS, Severe Acute Respiratory Syndrome.
(PDF)

S3 Fig The time dependence of *R*, *k*, and the proportion of cases causing superspreading events assuming trees are incomplete. (A) *R* decreased significantly between the first and second halves of transmission trees. (B) *k* increased significantly between the first and second halves of transmission trees. Seven of 39 trees had nonoptimizable degree distributions for the second half of the tree in each of 10 repetitions; these trees are excluded from this analysis and the boxplot. Y-axis is on a *log*_10_ scale for visual aid. (C) The proportion of cases causing superspreading events decreased significantly between the first and second halves of transmission trees. (D) While, on average, *R* decreased between first and second halves of trees, some trees had higher values of *R* in the second half of the tree than the first. Red line denotes *R* = 1. The Wilcoxon rank test was used for all significance tests (*: *p*≤0.05, **: *p*≤0.01, ***: *p*≤0.001, ****: *p*≤0.0001) and results are shown in red stars. Only trees with 20 or more cases and at least 2 generations of spread were used in these analyses. The data to reproduce this figure can be found at https://doi.org/10.5061/dryad.nk98sf7w7. COVID-19, Coronavirus Disease 2019; MERS, Middle East Respiratory Syndrome; SARS, Severe Acute Respiratory Syndrome.
(PDF)

S4 Fig Proportion of cases causing superspreading events and dispersion parameter estimates do not differ considerably with cutoff of 10 or more cases. (A) The highest proportion of cases causing superspreading events is observed at intermediate dispersion parameters, as predicted by theory [3]. (B) Dispersion parameter (*k*) of a negative binomial distribution fit to the offspring distribution of trees by disease (for diseases with at least 3 trees). Lower dispersion parameters are indicative of greater variation in number of secondary infections. Vertical line and value printed in each facet shows the median *k* and standard error for each disease. X-axes are on a *log*_10_ scale in both plots for visual aid. Only trees with 10 or more cases and at least 2 generations of spread were used in these analyses, and trees were assumed to be complete. The data to reproduce this figure can be found at https://doi.org/10.5061/dryad.nk98sf7w7. COVID-19, Coronavirus Disease 2019; MERS, Middle East Respiratory Syndrome; SARS, Severe Acute Respiratory Syndrome.
(PDF)

S5 Fig Proportion of cases causing superspreading events and dispersion parameter estimates do not differ considerably with cutoff of 30 or more cases, though fewer diseases are eligible for median dispersion parameter analysis. (A) The highest proportion of cases causing superspreading events is observed at intermediate dispersion parameters, as predicted by theory [3]. (B) Dispersion parameter (*k*) of a negative binomial distribution fit to the offspring distribution of trees by disease (for diseases with at least 3 trees). Lower dispersion parameters are indicative of greater variation in number of secondary infections. Vertical line and value printed in each facet shows the median *k* and standard error for each disease. X-axes are on a *log*_10_ scale in both plots for visual aid. Only trees with 30 or more cases and at least 2 generations of spread were used in these analyses, and trees were assumed to be complete. The data to reproduce this figure can be found at https://doi.org/10.5061/dryad.nk98sf7w7. COVID-19, Coronavirus Disease 2019; MERS, Middle East Respiratory Syndrome; SARS, Severe Acute Respiratory Syndrome.
(PDF)

S6 Fig Peak proportion of cases causing superspreading events is observed at a higher dispersion parameter (≈1), and dispersion parameter estimates are an order of magnitude higher when terminal nodes are excluded from dispersion parameter and *R* calculations than when terminal nodes are included. (A) The highest proportion of cases causing superspreading events is observed at intermediate dispersion parameters near 1, as opposed to the range of 0.2 to 0.6, as predicted by theory for higher values of R [3]. (B) Dispersion parameter (*k*) of a negative binomial distribution fit to the offspring distribution of trees by disease (for diseases with at least 3 trees). Lower dispersion parameters are indicative of greater variation in number of secondary infections. SARS now has the lowest median dispersion parameter of 0.87, mildly overdispersed. MERS, Ebola, and influenza would no longer be considered overdispersed. Vertical line and value printed in each facet shows the median *k* and standard error for each disease. X-axes are on a *log*_10_ scale in both plots for visual aid. Only trees with 20 or more cases and at least 2 generations of spread were used in these analyses. Terminal nodes were excluded from offspring distributions, i.e., trees were assumed to be incomplete. The data to reproduce this figure can be found at https://doi.org/10.5061/dryad.nk98sf7w7. COVID-19, Coronavirus Disease 2019; MERS, Middle East Respiratory Syndrome; SARS, Severe Acute Respiratory Syndrome.
(PDF)

S7 FigThere are too few trees with 2 or more superspreaders to examine superspreader dyads when R is calculated excluding terminal nodes.The expected number of superspreader-superspreader dyads was calculated by $\frac{s(s-1)}{S-t}$ for each tree, where *s* is the number of superspreaders in the tree, *t* is the number of terminal nodes, and *S* is tree size. Ratios larger than 1 indicate more superspreader-superspreader dyads observed than would be expected by chance. This analysis was limited to trees with more than 1 superspreader, 20 or more cases, and 2 or more generations of spread. The data to reproduce this figure can be found at https://doi.org/10.5061/dryad.nk98sf7w7. MERS, Middle East Respiratory Syndrome; SARS, Severe Acute Respiratory Syndrome.(PDF)

S1 Table The mean proportion of nodes with complete attribute information when that attribute was listed as available for a given tree. Analysis was limited to 5 most common attributes in the database and trees with 20 or more cases and 2 or more generations of spread.
(PDF)

## Abbreviations

COVID-19: Coronavirus Disease 2019
MERS: Middle East Respiratory Syndrome
SARS: Severe Acute Respiratory Syndrome
SARS-CoV: Severe Acute Respiratory Syndrome Coronavirus 1
