# Supplementary figures and images for: An open-access database of infectious disease transmission trees to explore superspreader epidemiology
Source: PLoS Biol. 2022 Jun 22;20(6):e3001685. doi: 10.1371/journal.pbio.3001685 (PMC9255728; doi:10.1371/journal.pbio.3001685)

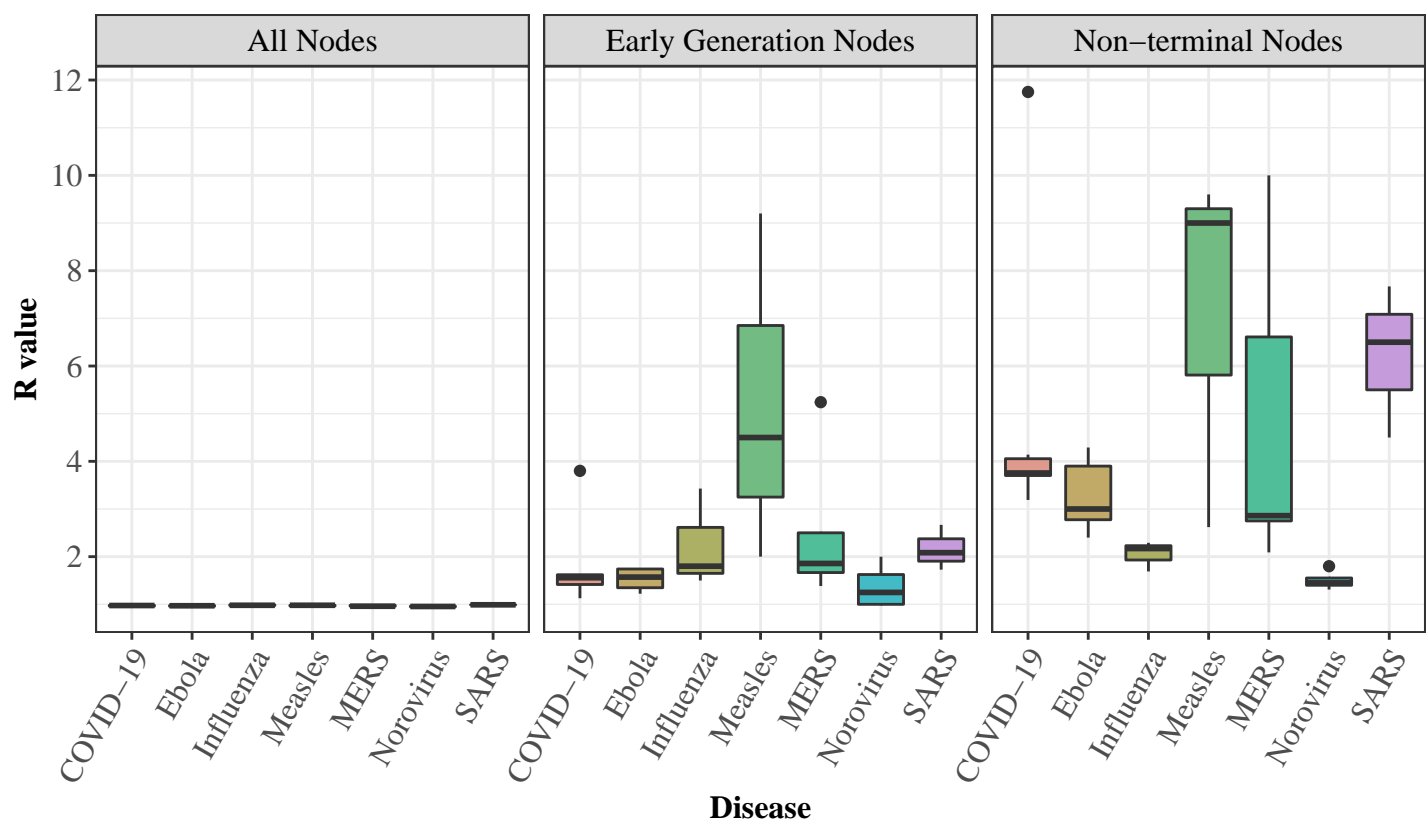

Supplement: S1 Fig — R values tended to be highest when calculated over nonterminal nodes and lowest when calculated over all nodes, with estimates based on early generation nodes (root and first generation nodes) falling somewhere in between. Nonterminal node estimates tended to be at the high end of literature values and early generation estimates at the low end, with estimates calculated over all nodes typically far below literature values [20,29,34–44], except for MERS and SARS which had low literature R estimates [3,21,30,45]. Analysis was limited to trees with 20 or more cases and at least 2 generations of spread and diseases with at least 3 trees that meet these criteria. The data to reproduce this figure can be found at https://doi.org/10.5061/dryad.nk98sf7w7. COVID-19, Coronavirus Disease 2019; MERS, Middle East Respiratory Syndrome; SARS, Severe Acute Respiratory Syndrome. (PDF) [file pbio.3001685.s002.pdf]

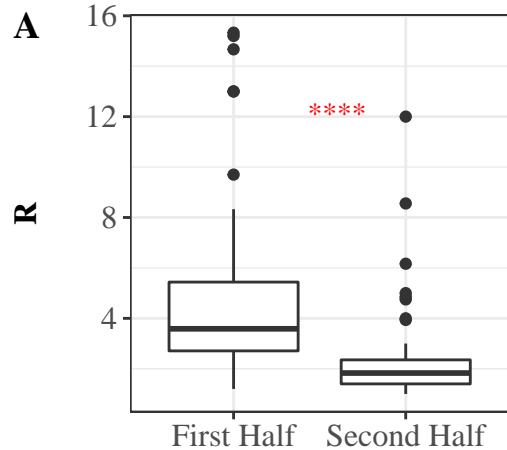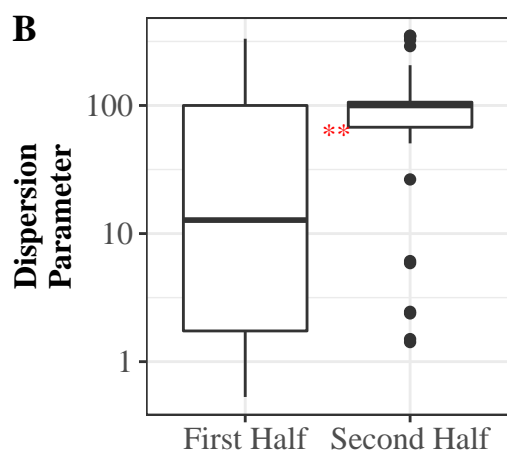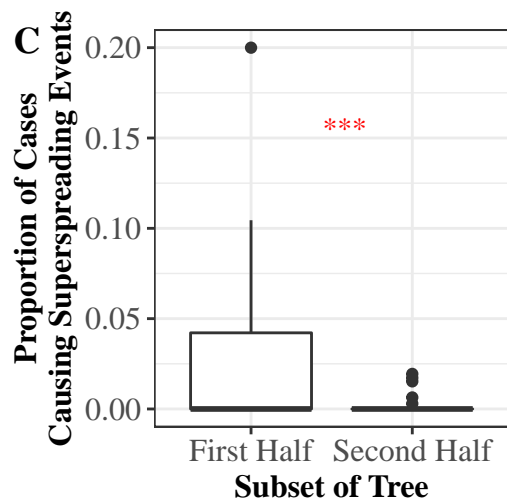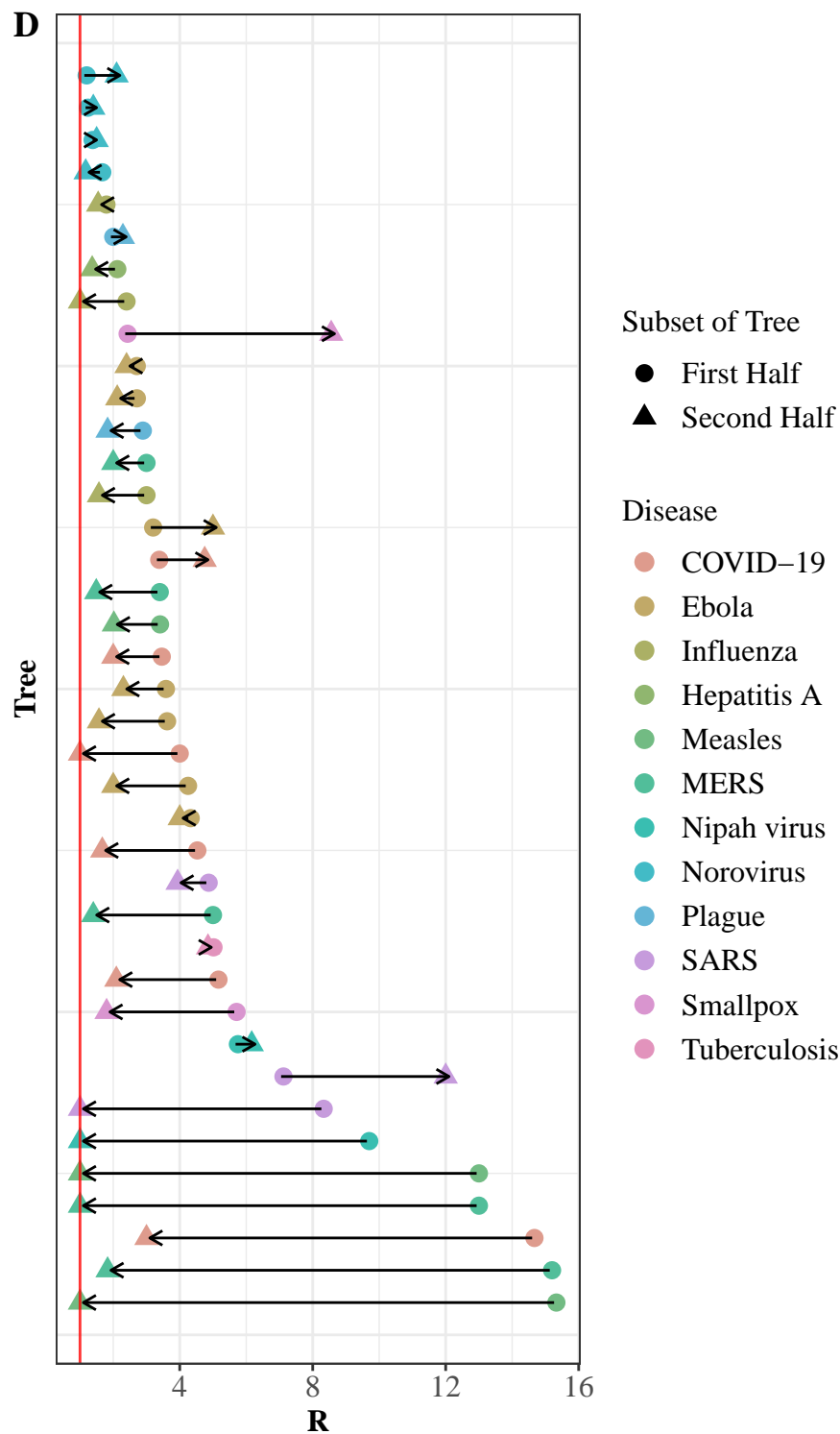

Supplement: S3 Fig — (A) R decreased significantly between the first and second halves of transmission trees. (B) k increased significantly between the first and second halves of transmission trees. Seven of 39 trees had nonoptimizable degree distributions for the second half of the tree in each of 10 repetitions; these trees are excluded from this analysis and the boxplot. Y-axis is on a log10 scale for visual aid. (C) The proportion of cases causing superspreading events decreased significantly between the first and second halves of transmission trees. (D) While, on average, R decreased between first and second halves of trees, some trees had higher values of R in the second half of the tree than the first. Red line denotes R = 1. The Wilcoxon rank test was used for all significance tests (*: p≤0.05, **: p≤0.01, ***: p≤0.001, ****: p≤0.0001) and results are shown in red stars. Only trees with 20 or more cases and at least 2 generations of spread were used in these analyses. The data to reproduce this figure can be found at https://doi.org/10.5061/dryad.nk98sf7w7. COVID-19, Coronavirus Disease 2019; MERS, Middle East Respiratory Syndrome; SARS, Severe Acute Respiratory Syndrome. (PDF) [file pbio.3001685.s004.pdf]

**Proportion of Cases Causing Superspreading Events**

**A**

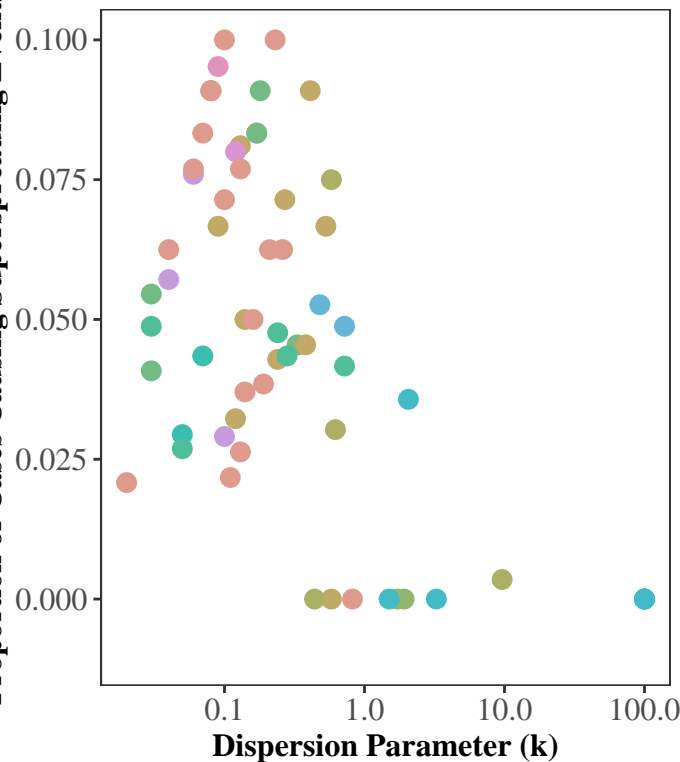

**Disease**

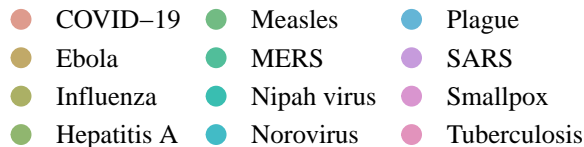

**B**

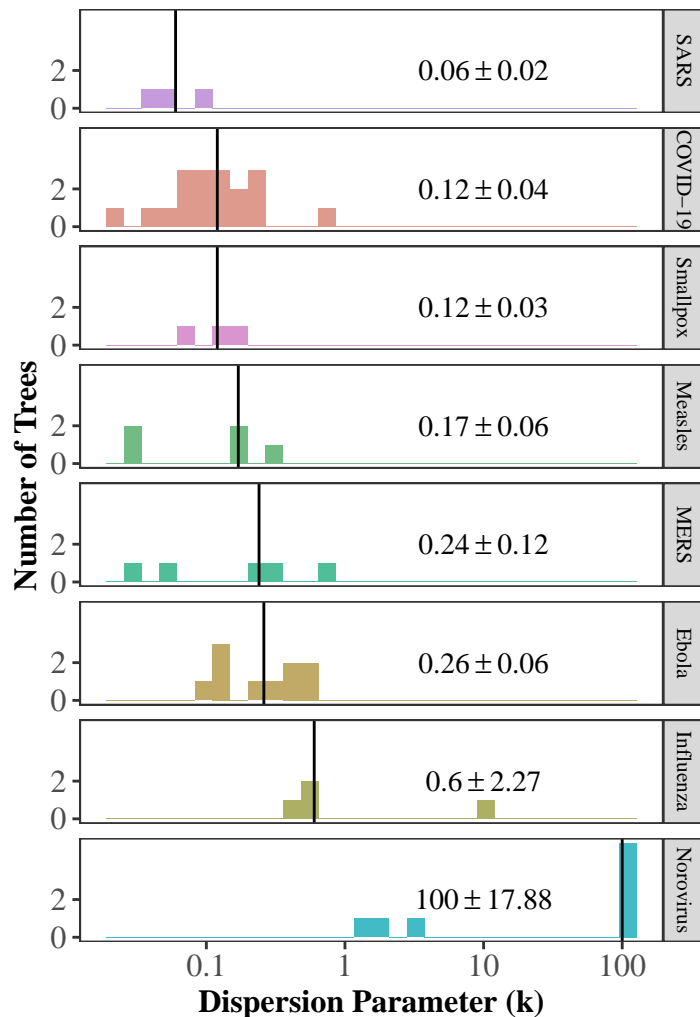

Supplement: S4 Fig — (A) The highest proportion of cases causing superspreading events is observed at intermediate dispersion parameters, as predicted by theory [3]. (B) Dispersion parameter (k) of a negative binomial distribution fit to the offspring distribution of trees by disease (for diseases with at least 3 trees). Lower dispersion parameters are indicative of greater variation in number of secondary infections. Vertical line and value printed in each facet shows the median k and standard error for each disease. X-axes are on a log10 scale in both plots for visual aid. Only trees with 10 or more cases and at least 2 generations of spread were used in these analyses, and trees were assumed to be complete. The data to reproduce this figure can be found at https://doi.org/10.5061/dryad.nk98sf7w7. COVID-19, Coronavirus Disease 2019; MERS, Middle East Respiratory Syndrome; SARS, Severe Acute Respiratory Syndrome. (PDF) [file pbio.3001685.s005.pdf]

Proportion of Cases Causing Superspreading Events

**A**

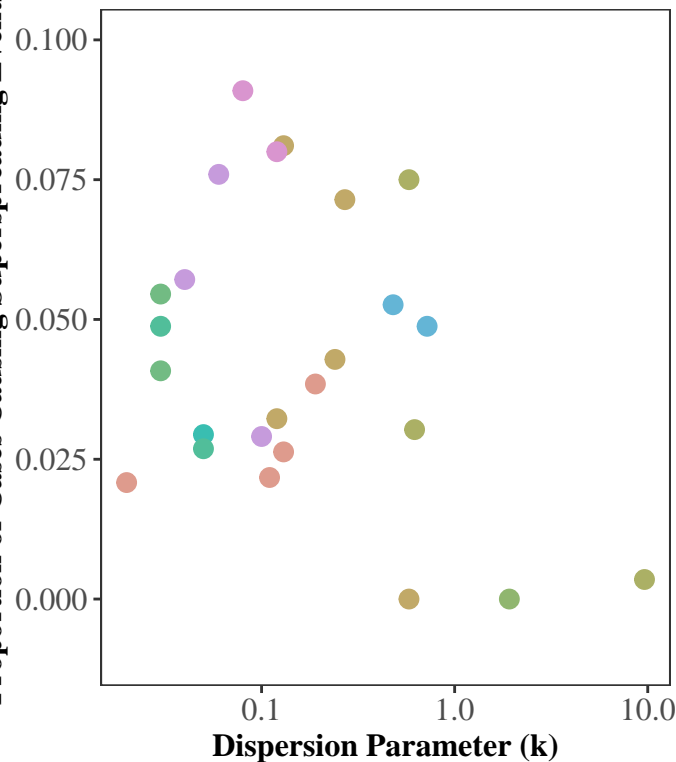

Disease

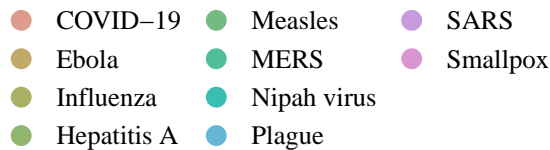

**B**

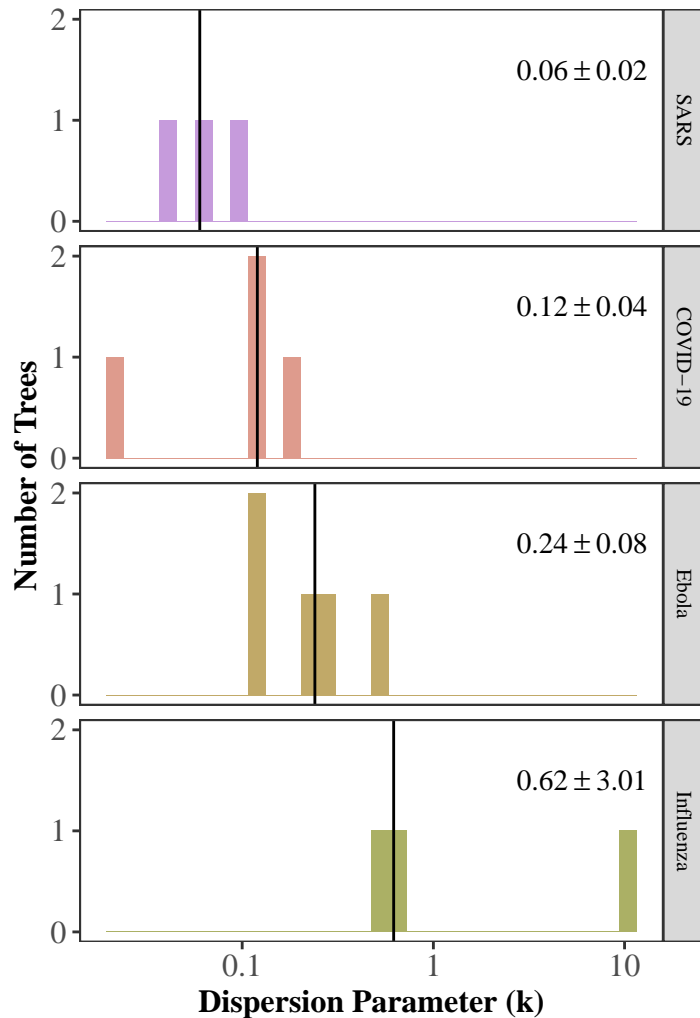

Supplement: S5 Fig — (A) The highest proportion of cases causing superspreading events is observed at intermediate dispersion parameters, as predicted by theory [3]. (B) Dispersion parameter (k) of a negative binomial distribution fit to the offspring distribution of trees by disease (for diseases with at least 3 trees). Lower dispersion parameters are indicative of greater variation in number of secondary infections. Vertical line and value printed in each facet shows the median k and standard error for each disease. X-axes are on a log10 scale in both plots for visual aid. Only trees with 30 or more cases and at least 2 generations of spread were used in these analyses, and trees were assumed to be complete. The data to reproduce this figure can be found at https://doi.org/10.5061/dryad.nk98sf7w7. COVID-19, Coronavirus Disease 2019; MERS, Middle East Respiratory Syndrome; SARS, Severe Acute Respiratory Syndrome. (PDF) [file pbio.3001685.s006.pdf]

**Proportion of Cases Causing Superspreading Events**

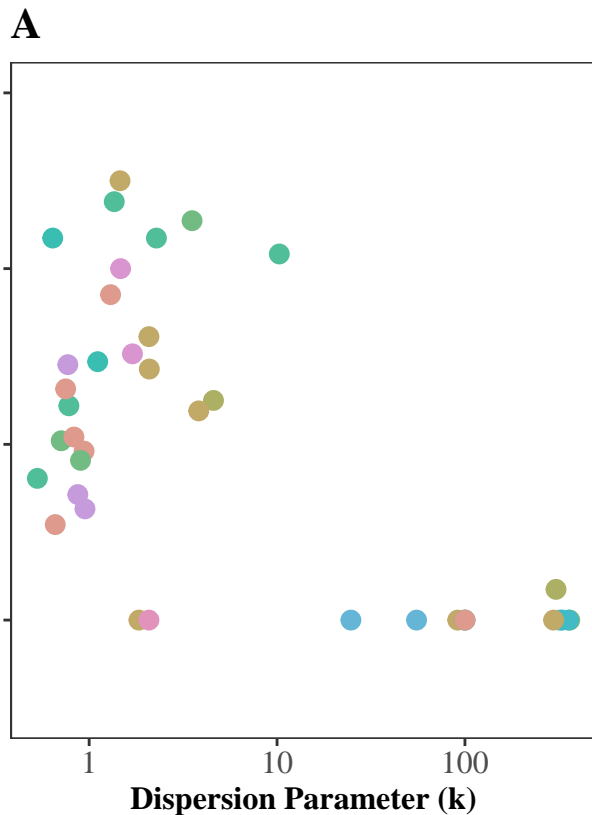

**Disease**

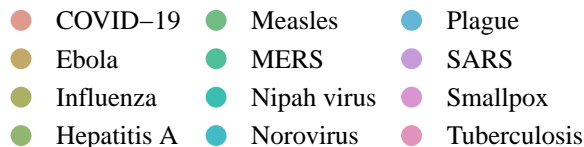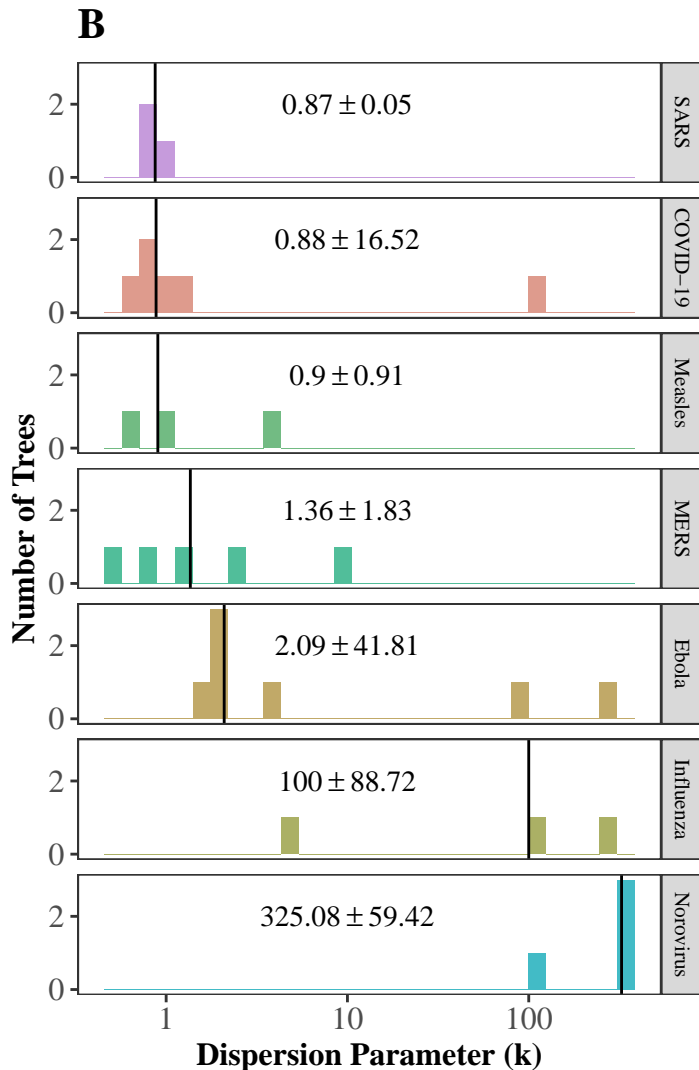

Supplement: S6 Fig — (A) The highest proportion of cases causing superspreading events is observed at intermediate dispersion parameters near 1, as opposed to the range of 0.2 to 0.6, as predicted by theory for higher values of R [3]. (B) Dispersion parameter (k) of a negative binomial distribution fit to the offspring distribution of trees by disease (for diseases with at least 3 trees). Lower dispersion parameters are indicative of greater variation in number of secondary infections. SARS now has the lowest median dispersion parameter of 0.87, mildly overdispersed. MERS, Ebola, and influenza would no longer be considered overdispersed. Vertical line and value printed in each facet shows the median k and standard error for each disease. X-axes are on a log10 scale in both plots for visual aid. Only trees with 20 or more cases and at least 2 generations of spread were used in these analyses. Terminal nodes were excluded from offspring distributions, i.e., trees were assumed to be incomplete. The data to reproduce this figure can be found at https://doi.org/10.5061/dryad.nk98sf7w7. COVID-19, Coronavirus Disease 2019; MERS, Middle East Respiratory Syndrome; SARS, Severe Acute Respiratory Syndrome. (PDF) [file pbio.3001685.s007.pdf]

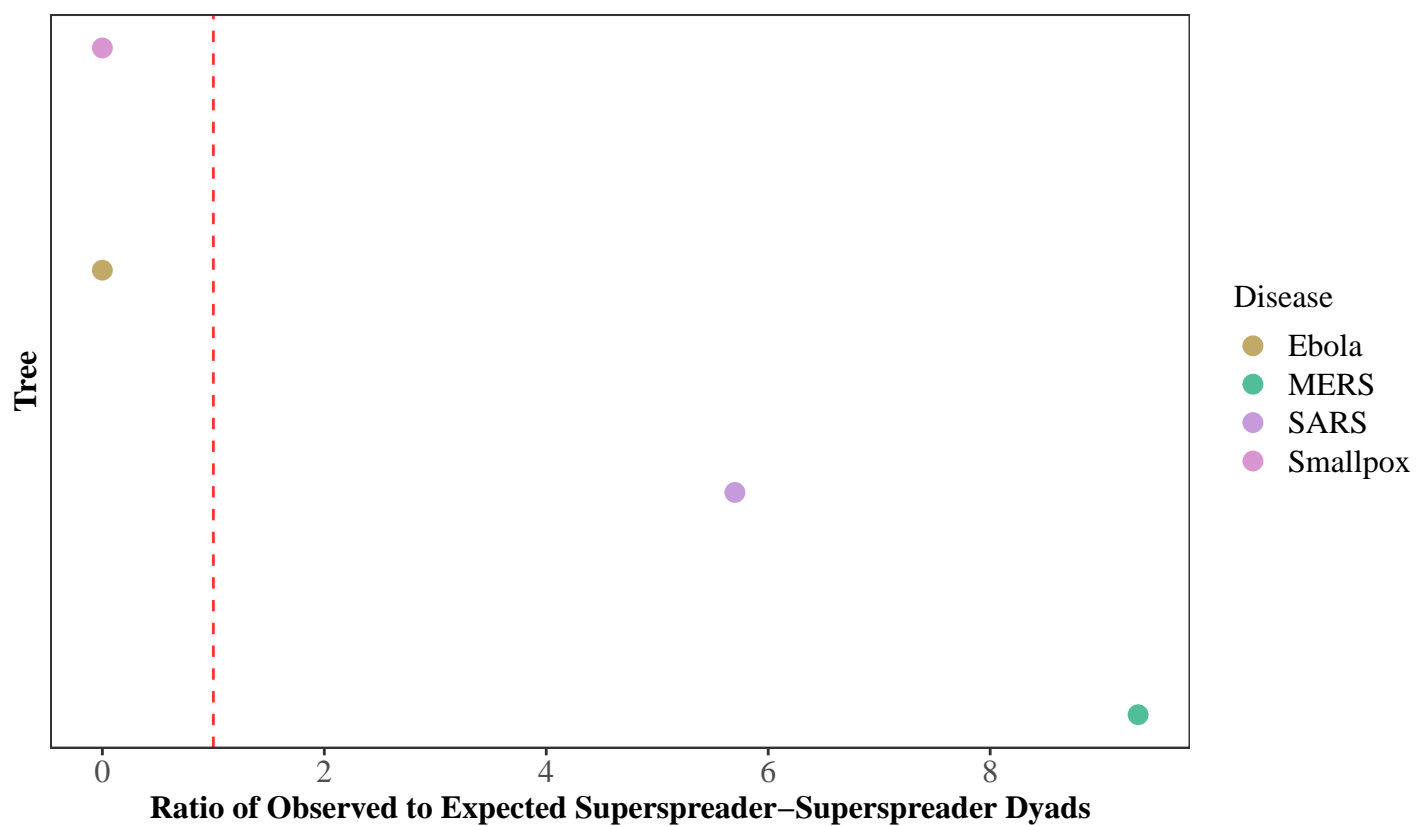

Supplement: S7 Fig — The expected number of superspreader-superspreader dyads was calculated by s(s−1)S−t for each tree, where s is the number of superspreaders in the tree, t is the number of terminal nodes, and S is tree size. Ratios larger than 1 indicate more superspreader-superspreader dyads observed than would be expected by chance. This analysis was limited to trees with more than 1 superspreader, 20 or more cases, and 2 or more generations of spread. The data to reproduce this figure can be found at https://doi.org/10.5061/dryad.nk98sf7w7. MERS, Middle East Respiratory Syndrome; SARS, Severe Acute Respiratory Syndrome. (PDF) [file pbio.3001685.s008.pdf]
