# Supplementary material for: An open-access database of infectious disease transmission trees to explore superspreader epidemiology
Source: PLoS Biol. 2022 Jun 22;20(6):e3001685. doi: 10.1371/journal.pbio.3001685 (PMC9255728; doi:10.1371/journal.pbio.3001685)
Supplement: S2 Fig — Dispersion parameter calculated over all nodes is on x-axis on log10 scale; dispersion parameter calculated over all nonterminal nodes is on y-axis on log10 scale. Dashed red line is y = x. Analysis was limited to trees with 20 or more cases and at least 2 generations of spread. The data to reproduce this figure can be found at https://doi.org/10.5061/dryad.nk98sf7w7. COVID-19, Coronavirus Disease 2019; MERS, Middle East Respiratory Syndrome; SARS, Severe Acute Respiratory Syndrome. (PDF) [file pbio.3001685.s003.pdf]

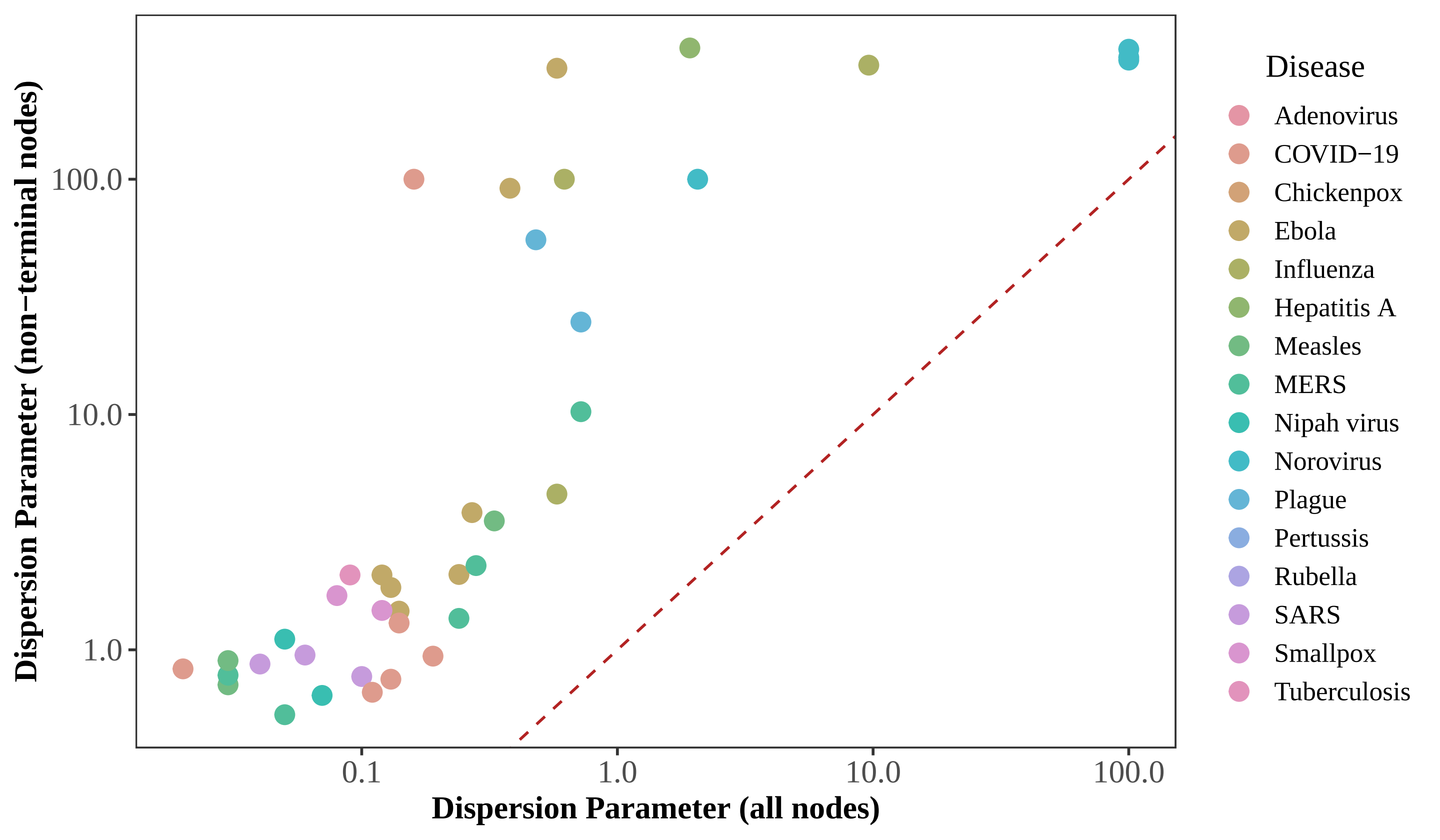

Dispersion Parameter (non−terminal nodes)
100.0
10.0
1.0
0.1
1.0
10.0
100.0
Dispersion Parameter (all nodes)
Disease
Adenovirus
COVID−19
Chickenpox
Ebola
Influenza
Hepatitis A
Measles
MERS
Nipah virus
Norovirus
Plague
Pertussis
Rubella
SARS
Smallpox
Tuberculosis
