# Supplementary material for: An open-access database of infectious disease transmission trees to explore superspreader epidemiology
Source: PLoS Biol. 2022 Jun 22;20(6):e3001685. doi: 10.1371/journal.pbio.3001685 (PMC9255728; doi:10.1371/journal.pbio.3001685)
Supplement: S1 Table — Analysis was limited to 5 most common attributes in the database and trees with 20 or more cases and 2 or more generations of spread. (PDF) [file pbio.3001685.s009.pdf]

Table 1: Mean proportion of individuals in a tree with complete attribute information. Trees limited to those with 20 or more cases and 2 or more generations of spread.

Attribute	Mean proportion
Transmission context	0.936
Symptom onset	0.822
Sex	0.740
Age	0.741
Location	0.919
